# Impact of Vitamin D supplementation on cognition in adults with mild to moderate vitamin D deficiency: Outcomes from the VitaMIND randomised controlled trial

**DOI:** 10.1002/alz70860_105178

**Published:** 2025-12-23

**Authors:** Anne Corbett, Rod Taylor, David J Llewellyn, Janice M Ranson, Adam Hampshire, Ellie Pickering, Abbie Palmer, Dag Aarsland, Dorina Cader, Diana Frost, Clive G Ballard

**Affiliations:** ^1^ University of Exeter, Exeter, United Kingdom; ^2^ Glasgow University, Glasgow, Scotland, United Kingdom; ^3^ University of Exeter, Exeter, Devon, United Kingdom; ^4^ Imperial College London, Department of Brain Sciences, London, London, United Kingdom; ^5^ Institute of Psychiatry, Psychology & Neuroscience, King's College London, London, United Kingdom; ^6^ Brighton and Sussex Medical School, Brighton, Brighton, United Kingdom; ^7^ University of Exeter, Exeter, Exeter, United Kingdom

## Abstract

**Background:**

Preserved cognitive health with ageing is a public health imperative. Vitamin D deficiency is associated with poor cognition, but it is unclear whether supplementation would provide benefit, particularly in individuals with mild/moderate deficiencies which do not have other clinical risks. The objective of this study was to establish the impact of daily vitamin D supplementation on cognition in older adults with mild to moderate vitamin D deficiency.

**Method:**

This was a two‐arm parallel 24‐month randomised controlled trial, with Vitamin D supplementation compared with a placebo. It was a remote trial, completed from home involving 620 adults, aged 50 years or older, with mild to moderate vitamin D deficiency and early cognitive impairment. The primary outcome was executive function measured through Trail making B and other secondary measures of cognition, function and wellbeing.

**Result:**

Vitamin D supplementation conferred no significant benefit to executive function compared to placebo at follow‐up on the primary outcome (between‐group difference: 5770, 95% CI: ‐2189 to 13730) or cognition, function, or wellbeing. Secondary analyses in defined subgroups and a per‐protocol analysis also showed no significant impact on any outcome measures.

**Conclusion:**

Vitamin D supplementation produced no measurable improvement in cognitive outcomes in older adults with mild to moderate vitamin D deficiency. The remote trial methodology provides an innovative approach to large‐scale trials and should be considered for low‐risk nutraceutical or repositioned drug trials in the future.

